# Gas Phase Fragmentation Behavior of Proline in Macrocyclic *b*_7_ Ions

**DOI:** 10.1021/jasms.3c00049

**Published:** 2023-07-04

**Authors:** Cagdas Tasoglu, Alper Arslanoglu, Talat Yalcin

**Affiliations:** † National Mass Spectrometry Application and Research Center, Integrated Research Centers, Izmir Institute of Technology, Urla-Izmir 35430, Turkey; ‡ Department of Molecular Biology and Genetics, Izmir Institute of Technology, Urla-Izmir 35430, Turkey; § Department of Chemistry, Faculty of Science, Izmir Institute of Technology, Urla-Izmir 35430, Turkey

**Keywords:** proline, peptide fragmentation, scrambling of sequence, nondirect sequence ions, macrocyclization, ESI MS/MS mass spectrometry

## Abstract

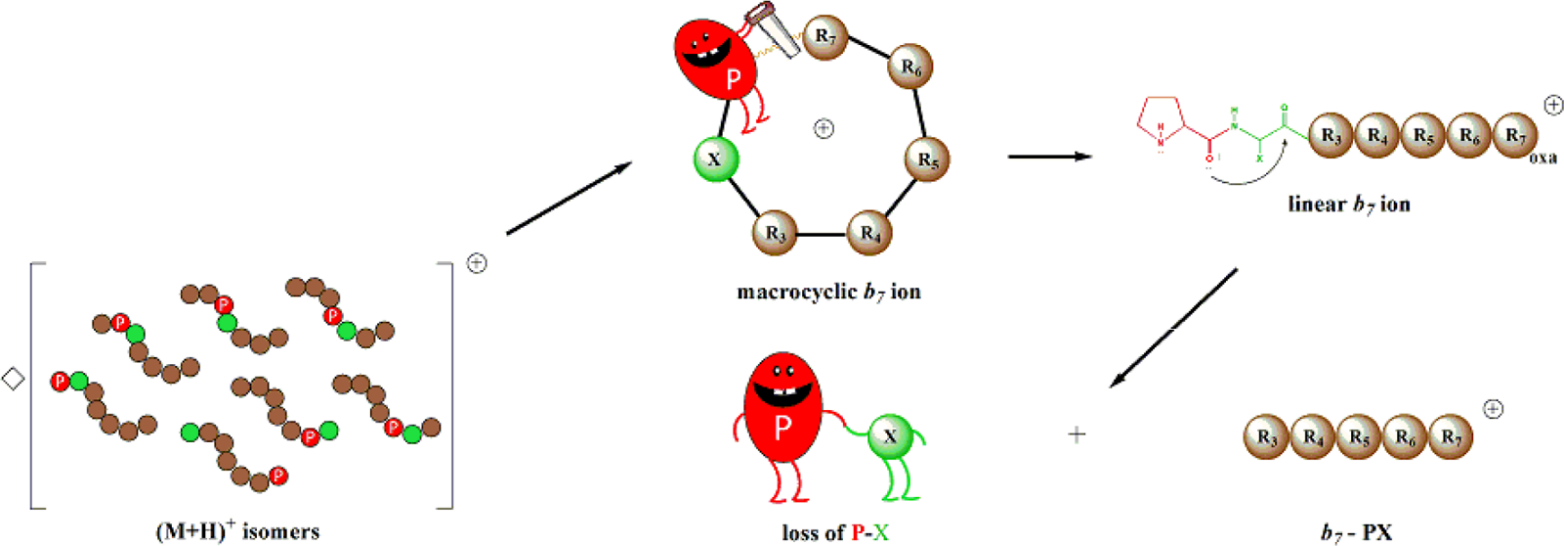

The fragmentation characteristics of *b*_7_ ions produced from proline-containing heptapeptides have been studied in detail. The study has utilized the following C-terminally amidated model peptides: PA_6_, APA_5_, A_2_PA_4_, A_3_PA_3_, A_4_PA_2_, A_5_PA, A_6_P, PYAGFLV, PAGFLVY, PGFLVYA, PFLVYAG, PLVYAGF, PVYAGFL, YPAGFLV, YAPGFLV, YAGPFLV, YAGFPLV, YAGFLPV, YAGFLVP, PYAFLVG, PVLFYAG, A_2_PXA_3_, and A_2_XPA_3_ (where X = C, D, F, G, L, V, and Y, respectively). The results have shown that *b*_7_ ions undergo head-to-tail cyclization and form a macrocyclic structure. Under the collision-induced dissociation (CID) condition, it generates nondirect sequence ions regardless of the position of the proline and the neighboring amino acid residues. This study highlights the unusual and unique fragmentation behavior of proline-containing heptapeptides. Following the head-to-tail cyclization, the ring opens up and places the proline residue in the N-terminal position while forming a regular oxazolone form of *b*_2_ ions for all peptide series. Then, the fragmentation reaction pathway is followed by the elimination of proline with its C-terminal neighbor residue as an oxazolone (e.g., PX_oxa_) for all proline-containing peptide series.

## Introduction

Due to its imino group being held in a stiff confirmation and lowering the structural flexibility of the polypeptide chain, proline amino acid plays a significant role in the stability of proteins.^1^ Therefore, it is not surprising that 99.8% of human proteins analyzed contain proline. In comparison, certain proteins have up to 40% proline residues,^2^ and correct identification of proline-containing peptides by mass spectrometric methods is important for proteomic studies.

In the last two decades, tandem mass spectrometry (MS^n^) with collision-induced dissociation (CID) has become an indispensable analytical technique used for protein/peptide sequencing^3,4^ with the emergence of soft ionization methods: electrospray ionization (ESI)^5,6^ and matrix-assisted laser desorption/ionization (MALDI).^7,8^ Low-energy CID conditions lead to cleavages at the peptide backbone and mainly produce sequence-informative *a*, *b*, and *y* ions^9,10^ through charge-directed reactions (mobile proton model).^11^ Theoretical *m*/*z* data of enzymatic peptides from known proteins are matched to those obtained for product ions to identify the protein. Mascot^12^ and SEQUEST^13^ are popular matching algorithms that utilize simple and primitive peptide fragmentation chemistry. This limitation, in turn, may result in erroneous assignments in protein identification.

While *y* ions were established to be truncated peptides,^14,15^ acylium ion was suggested as a structure of *b* ion by early publications.^9,10^ In contrast, it was Boyd’s group first proposing the formation of the cyclic structure of doubly protonated *b* ions.^16,17^ In addition, Yalcin et al.^18^ revealed that cyclization occurs via nucleophilic attack of adjacent carbonyl oxygen on the N-terminus to generate a five-membered oxazolone ring structure of *b* ions. This mechanism was also verified by the infrared multiple photon dissociation (IRMPD) techniques^19−23^ and deuterium labeling experiments.^24^ Diketopiperazine was then alternatively proposed as the structure of *b*_2_ ions by Wesdemiotis and co-workers.^15,25^ In contrast, Wysocki et al.^26^ reported that the structure of *b*_2_ ions produced from the dipeptide of HA is a mixture of oxazolone and diketopiperazine. It was also shown that the cyclic structure of *b*_*n*_ ion (*n* = 2 or 4) is peptide-side-chain-dependent.^27−34^ However, *b*_*n*_ ions (*n* ≥ 5) formed a macrocyclic structure via head-to-tail cyclization. This macrocyclic *b*_*n*_ ion randomly breaks apart and forms “*nondirect sequence ions*” and “*direct sequence ions*” upon CID conditions^35−38^ CID condition.

Proline has the highest proton affinity among amino acid residues without basic functional groups.^39^ Many studies^3,40−46^ demonstrated proline-directed fragmentations that produce very prominent *y* ions due to the cleavage of N-terminal to proline residue (proline effect). The position of proline was also found to have a direct role at the cleavage site of protonated peptides^47^ and deprotonated ones, too.^48^ Eckart’s group published the first experimental and theoretical results of an alternative gas-phase structure for the *b*_2_ ion (immonium ion type of *b*_2_) produced from GP.^49^ Wysocki’s laboratory suggested a diketopiperazine structure for *b*_2_ ion obtained from VP *b*_2_ ion;^50,51^ however, then they observed predominant oxazolone structures of *b*_2_ ion structure for PPG tripeptide^52^ and concluded that the neighbor residue has an influence on the *b*_2_ ion structure because Martens et al.^53^ found earlier that the diketopiperazine pathway is almost 100% preferred for the *b*_2_ ion of PPP.

Here we investigate the gas phase fragmentation behavior of a proline-containing heptapeptide series in the gas phase. We observe a unique fragmentation pathway eliminating proline with its C-terminal neighbor residue as a neutral oxazolone (PX_oxa_). MS/MS results show the formation of proline-containing macrocyclic *b*_7_ ions, followed by ring opening (linearization), rearrangement of the proline residue at the N-terminal position, and then the elimination of proline together with its C-terminal neighbor residue as a neutral oxazolone. To our knowledge, such elimination of oxazolone is the first experimental observation for the proline-containing heptapeptide series.

## Experimental Section

### Materials

All synthetic model peptides were obtained from GL Biochem and used as received with no further purification. HPLC-grade methanol and formic acid were supplied by Merck (Darmstadt, Germany). The water was ultrapure grade (Arium 611 UV, Sartorius AG, Goettingen, Germany). Stock solutions of peptides were prepared by dissolving solid material in a 1:1 (v/v) mixture of methanol and water to a concentration of 10^–2^ M. Peptide samples at micromolar concentration level were prepared by diluting stock solutions with 1:1 methanol:water containing 1% formic acid. All of the model peptides are C-terminally amidated except for YIHPFHL-OH.

### Mass Spectrometry

All low-energy CID experiments were conducted on an LTQ XL linear ion trap mass spectrometer (Thermo Scientific, NJ, USA), equipped with an electrospray ionization source. Peptide samples were infused into an electrospray source at a flow rate of 5 μL/min using an incorporated syringe pump.

The linear ion trap MS analyses were performed under the following conditions: All of the instrumental parameter settings were optimized to get maximum precursor ion transmission to the ion trap mass analyzer using the autotune routine within the LTQ Tune program. Ion spray voltage was kept at 5.00 kV, and nitrogen was used as a sheath gas (10 au), auxiliary gas (1 au), and sweep gas (1 au) for spray stabilization. Ion-trapping efficiency and collisional cooling were improved by using helium as a bath gas that was also used as a collision (target) gas for CID analysis. The capillary temperature was held constant at 275 °C. Multistage mass (MS^n^) analysis was carried out using an isolation width of 2.1 *m*/*z*, an activation time of 30 ms, and a time of acquisition of 0.30 min. The normalized collision energy was maintained between 23% and 27% for all CID experiments.

## Results and Discussion

### Fragmentation Reactions of *b*_7_ Ions from Isomers of PA_6_

**Figure 1:**
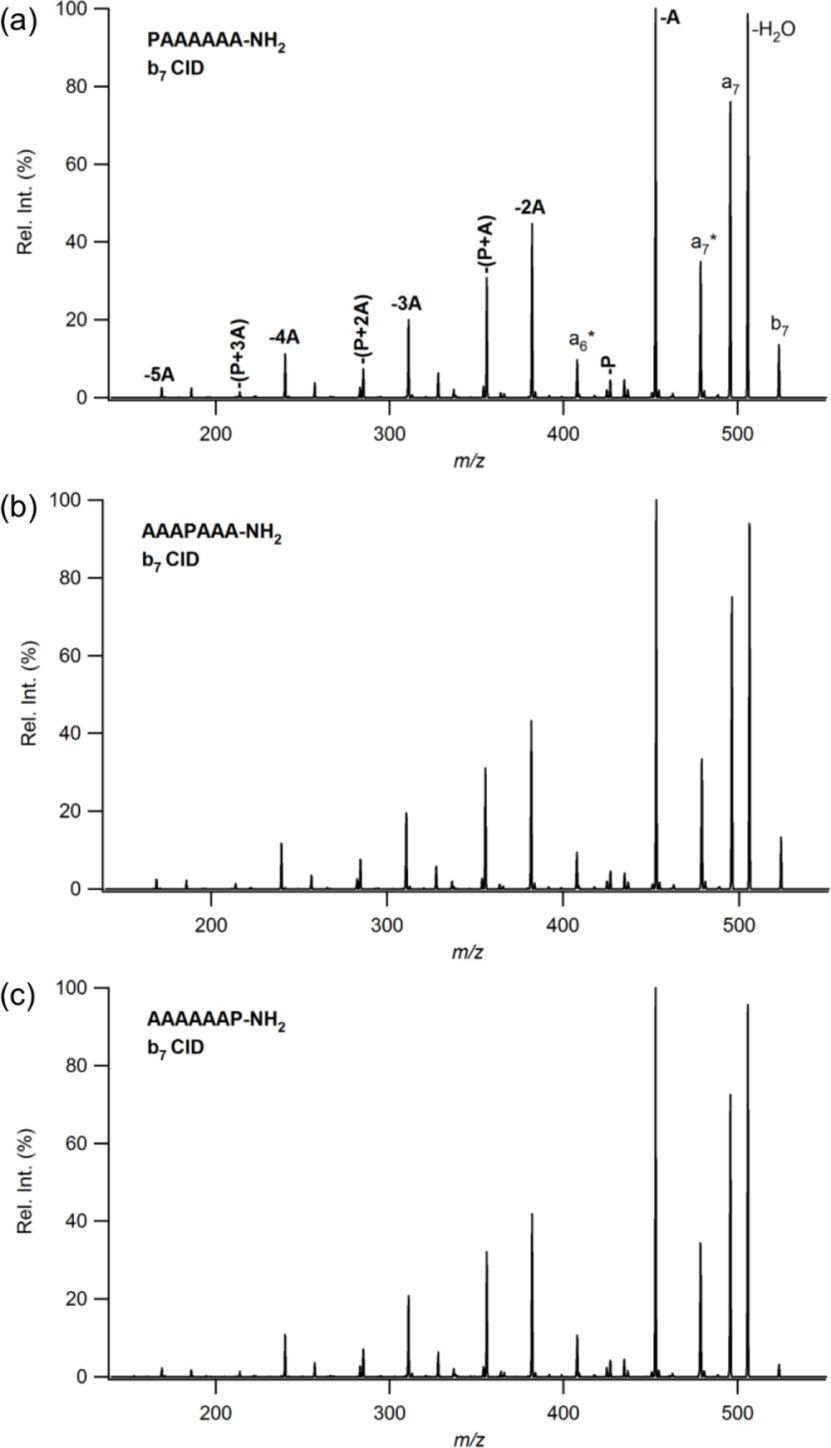
MS/MS spectra of *b*_7_ ions derived from protonated (a) PAAAAAA-NH_2_, (b) AAAPAAA-NH_2_, and (c) AAAAAAP-NH_2_.

MS/MS spectra of *b*_7_ (524 *m*/*z*) ions produced from protonated heptapeptides (containing six alanine (A) residues and a single proline (P) residue) are given in Figure 1. Nearly identical fragmentation patterns are obtained for all of the isomers. In addition to direct sequence ions of H_2_O loss (506 *m*/*z*), *a*_7_ (496 *m*/*z*), and *a*_7_* (479 *m*/*z*), we also observe nondirect sequence ions as eliminations of A (453 *m*/*z*), 2A (382 *m*/*z*), 3A (311 *m*/*z*), 4A (240 *m*/*z*), 5A (169 *m*/*z*), P (427 *m*/*z*), P+A (356 *m*/*z*), P+2A (285 *m*/*z*), and P+3A (214 *m*/*z*). MS/MS spectra of *b*_7_ ions of APAAAAA-NH_2_, AAPAAAA-NH_2_, AAAAPAA-NH_2_, and AAAAAPA-NH_2_ isomers also produce the same product ions and fragmentation patterns (Figure S1). The obtained results are in good agreement with the work of Harrison,^54^ showing identical MS/MS spectra of *b*_5_ ions derived from PAAAA, AAPAA, and AAAAP series. Internal eliminations and similarity of dissociation patterns are the direct evidence of a head-to-tail macrocyclization reaction before reopening at various amide bond positions regardless of the original sequence. These findings clearly show that the position of proline does not influence the course of fragmentation for the alanine-containing heptapeptide series.

### Fragmentation Reactions of *b*_7_ Ions from Isomers of PYAGFLV

**Figure 2:**
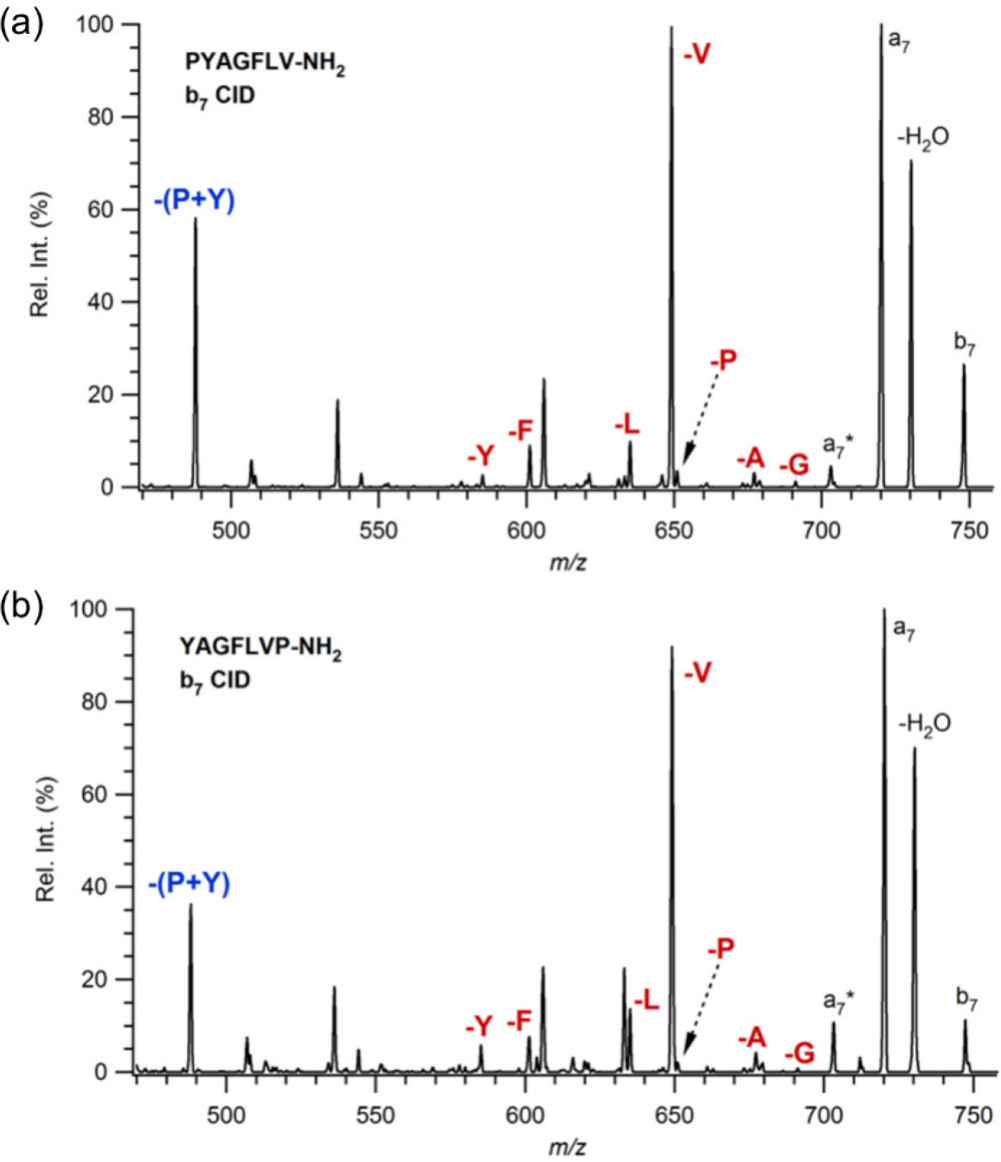
MS/MS spectra of *b*_7_ ions derived from protonated (a) PYAGFLV-NH_2_ and (b) YAGFLVP-NH_2_.

**Figure 3:**
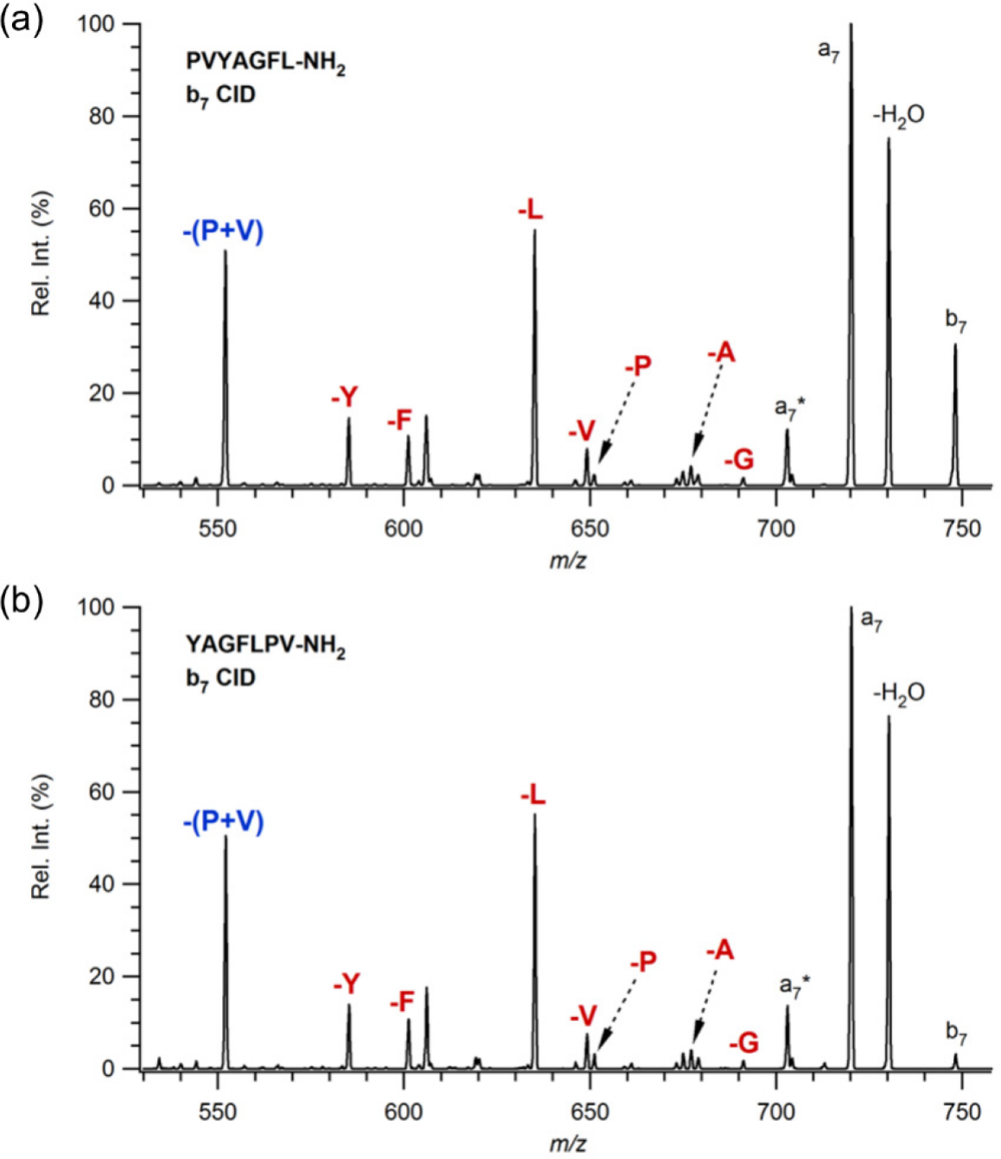
MS/MS spectra of *b*_7_ ions derived from protonated (a) PVYAGFL-NH_2_ and (b) YAGFLPV-NH_2_.

The MS/MS spectra of *b*_7_ ions produced from PYAGFLV-NH_2_ and its other isomers are compared to determine the influence of the neighbor residue and the position of proline on the fragmentation pathway. Figure 2 shows the MS/MS spectra of *b*_7_ ions produced from PYAGFLV-NH_2_ (a) and YAGFLVP-NH_2_ (b) in the high mass range. Similar MS/MS spectra are observed for all isomeric heptapeptide series. The most abundant ions in the mass spectra were H_2_O loss at *m*/*z* 730, *a*_7_ ions at *m*/*z* 720, and *b*_7_-V at *m*/*z* 649. In addition, internal single residue eliminations such as *b*_7_-X ions (where X is P, Y, A, G, F, or L) corresponding to *m*/*z* 651, 585, 677, 691, 601, and 635, respectively, are observed. What is noticeable is abundant proline, P, and tyrosine, Y, residue elimination, together from the *b*_7_ ion for both isomers. The single P elimination from *b*_7_ is not a preferred route; instead, the loss of P and Y residues is observed as the favored pathway. Similar behavior is also observed for the isomeric pair of PVYAGFL and YAGFLVP, where P and valine, V, residue loss is abundant in the MS/MS spectra. At the same time, single eliminations of each residue are also observed and are shown in Figure 3a and b.

Further experiments are carried out for the isomeric pairs of PLVYAGF/YAGFPLV, PAGFLVY/YPAGFLV, PGFLVYA/YAPGFLV, and PFLVYAG/YAGPFLV. The MS/MS spectra of *b*_7_ ions produced from these isomeric peptide series show a similar fragmentation pathway, and always *b*_7_-PX residue elimination is observed for each isomer (where X is L, A, G, and F, and it is a C-terminally connected residue to P). The results are shown in Figures S2–S5. Because each pair has the same ring sequence when macro-cycled, it can be concluded that the original position of the proline does not affect the fragment ion distribution and course of fragmentation.

Contrary to abundant PY and PV residue eliminations, a minor proline-phenylalanine (PF) elimination is observed from *b*_7_ ions produced from the PFLVYAG/YAGPFLV peptide pair (Figure S5). Since the glycine residue stays N-terminal to the proline residue when PFLVYAG and YAGPFLV are macro-cycled, it is conceivable to consider that the glycine residue is the reason for the low abundance of PF elimination. The MS/MS spectra of *b*_7_ ions produced from PYAFLVG and PVLFYAG show similar behavior where the elimination of PY and PV residues from *b*_7_ ions is very low (Figure S6). Thus, it can be concluded that mobile protons would not prefer to be retained at the G–P amide bond due to glycine’s low proton affinity. Thus, the intense proline-X residue elimination from *b*_7_ ions significantly decreases when the glycine is N-terminal to proline. The glycine effect is well-known in the peptide fragmentation mechanism.^38,43,55^

**Figure 4:**
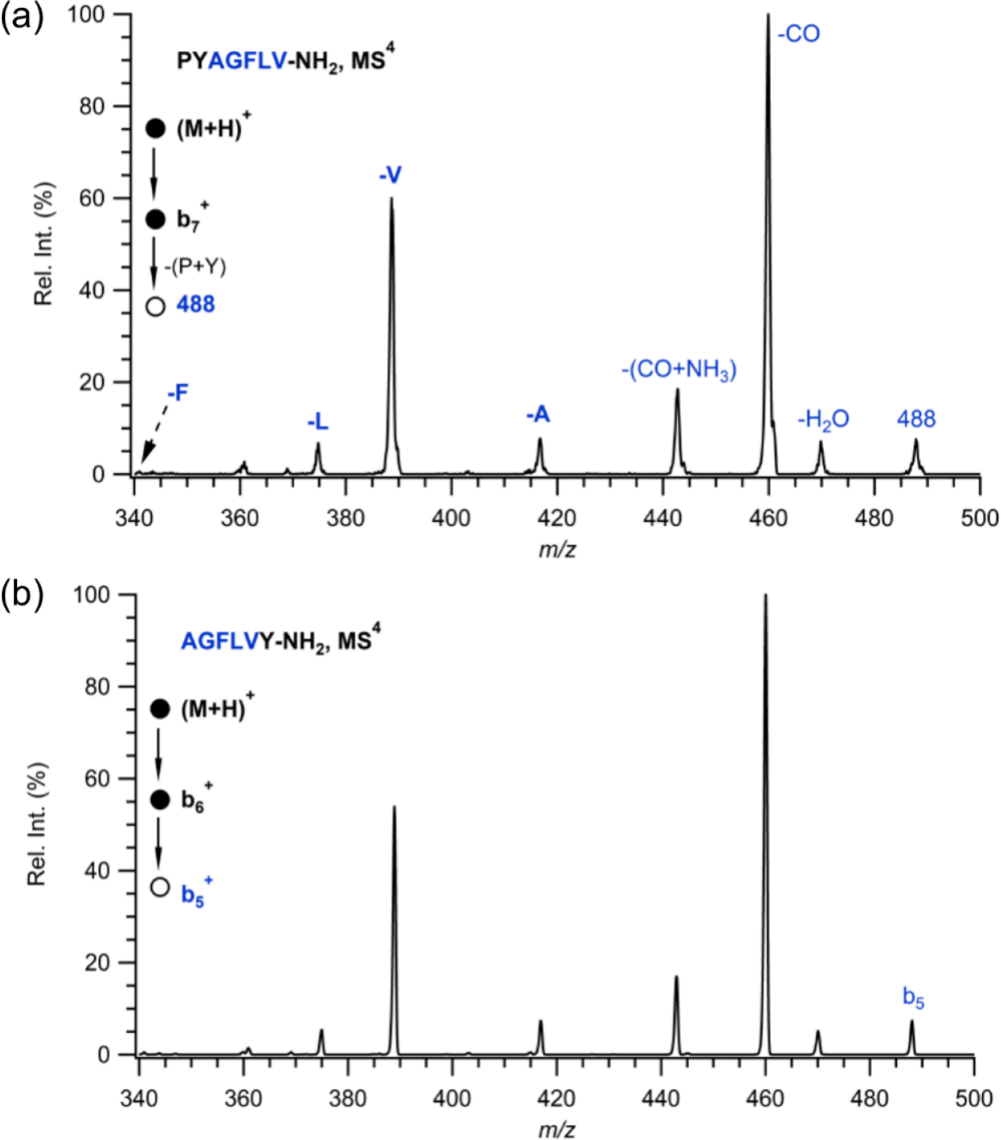
Comparison of MS^4^ spectra of (a) P–Y residue elimination from *b*_7_ of protonated PYAGFLV-NH_2_ and (b) *b*_5_ of protonated AGFLVY-NH_2_.

The MS^4^ experiments are performed for the *b*_7_-PY residue (nominally *b*_5_) and *b*_5_ ion produced from AGFLVY to confirm the remaining amino acid sequence after the PY residue loss from the *b*_7_ ion produced from PYAGFLV. The MS/MS spectra of the remaining peptide after PX residue elimination from the *b*_7_ ion are shown in Figure 4. The MS/MS spectra of *b*_7_-PY ions produced from PYAGFLV and the MS/MS spectra of *b*_5_ ions produced from AGFLVY are precisely the same as expected. Similarly, other fragment pairs in Table 1 also produce the same pattern (Figures S7–S11). The PX residue elimination observed from *b*_7_ ions is a unique fragmentation behavior of heptapeptides containing a proline residue. The MS/MS spectra of all isomer pairs show similar fragmentation pathways such as the elimination of H_2_O, CO, and CO+NH_3_. In addition, single residue eliminations indicate the macrocyclization/reopening pathway for the remaining pentapeptide.

**Table 1:**
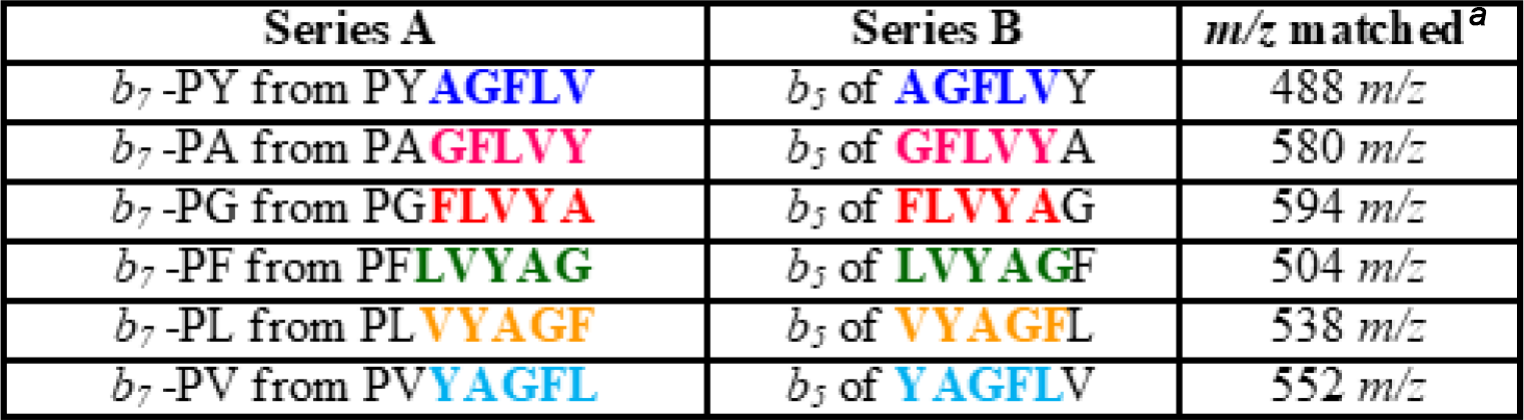
Comparison of Peptide Fragments to Prove the Identity of the P–X Residue Elimination^a^

a The term “*m*/*z* matched” corresponds to the mass of the common colored sequence of compared peptides in each series.

The behavior of proline residues is further studied using model peptides YAGHFLV and YAGKFLV, where P is replaced with other amino acids such as histidine (H) and lysine (K). The CID of *b*_7_ of YAGHFLV does not result in the loss of H and its C-terminal residue F, while *b*_7_ of YAGKFLV shows a minor K+F elimination with approximately 3% intensity (Figure S12a and b). Additionally, 26% K+G elimination is observed from the *b*_7_ ions of the same peptides, further confirming the specificity of our findings to proline residues.

To determine if the behavior of proline residues is consistent in the presence of a more basic residue, we examined another peptide model, YIHPFHL-OH (Figure S12c). We observe abundant elimination of P+F from *b*_7_, but unexpectedly, P+H elimination was also observed, most likely due to the higher basicity of the H residue. Nonetheless, this observation does not contradict our finding that P and its C-terminal neighbor residue are neutrally eliminated from *b*_7_ ions.

### Fragmentation Reactions of *b*_7_ Ions from AAPXAAA and AAXPAAA (Where X Is C, D, F, G, L, V, and Y)

**Figure 5:**
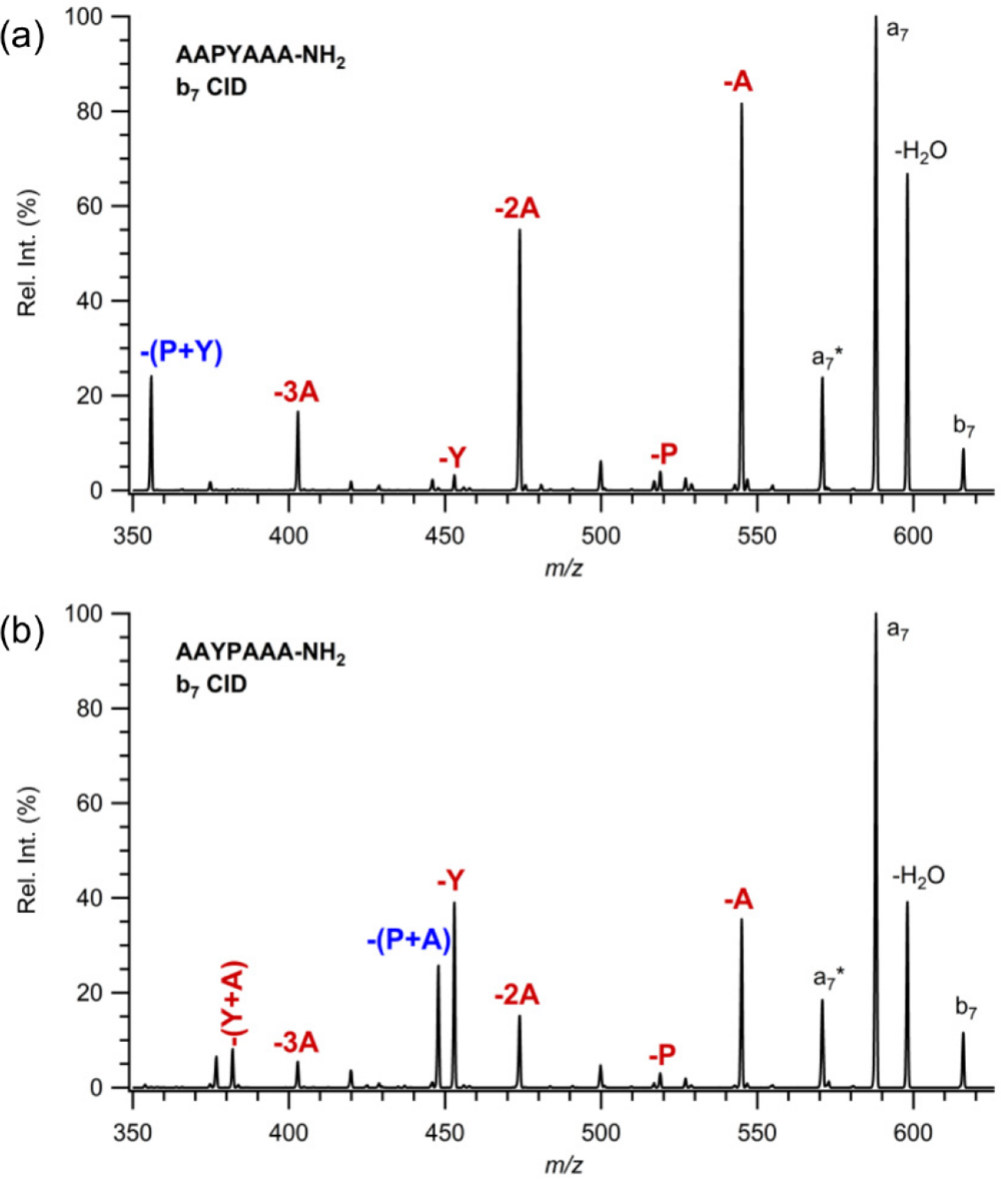
Comparison of MS/MS spectra of *b*_7_ from protonated (a) AAPYAAA-NH_2_ and (b) AAYPAAA-NH_2_.

A different set of model peptides, such as AAPXAAA and AAXPAAA, where X is C, D, F, G, L, V, and Y residues, is investigated to gather information about PX or XP residue elimination from *b*_7_ ions. The MS/MS spectra of *b*_7_ ions produced from AAPYAAA and AAYPAAA are shown in Figure 5a and b. As expected, an abundant elimination of the PY residue is observed from *b*_7_ ions produced from AAPYAAA. By contrast, PA residue elimination is abundant in the MS/MS spectra of *b*_7_ ions produced from AAYPAAA. This result is consistent with our findings suggesting that proline and its adjacent C-terminal residue are lost as a neutral PX residue regardless of their position in the peptide backbone. This behavior is well-supported by increased Y elimination from *b*_7_ when Y is N-terminal to the P residue (Figure 5b). The same fragmentation pathway is observed for other model peptides where X is C, D, F, G, L, and V (Figures S13–S18). Abundant single elimination of amino acid residues N-terminal to proline can be explained by a propensity of proline to stay at the N-terminus after macrocyclization and reopening reaction. And then, the related residue can be located at the C-terminus to be eliminated as a single residue (direct sequence pathway). However, this behavior is not favored when the X amino acid residue is replaced by P. In this case, negligible PP residue elimination is seen (∼0.6%) in *b*_7_ spectra of PPAAAAA, AAPPAAA, and AAAAAPP peptide series (Figure S19). This behavior is most likely due to the proline’s unique rigid cyclic structure, where the N-terminal proline’s carbonyl group cannot effectively attack the carbonyl group of the neighboring proline for the formation and elimination of neutral oxazolone. The unique structure of PP and its enhanced basicity may be attributed to basic functional groups, such as amino groups, which can readily accept protons. PP is a cyclic dipeptide that contains two proline residues, which are known to have unique structural characteristics that distinguish them from those of other amino acids. For example, proline contains a rigid cyclic structure due to its side chain being connected to the backbone nitrogen atom, which can influence the overall conformation of a protein or peptide. Additionally, the presence of multiple nitrogen atoms in proline residues can increase the basicity of the molecule, enhancing its propensity to accept protons. This combination of unique structural features and enhanced basicity may contribute to the distinct behavior of PP in various chemical and biological contexts.^46,56^

**Scheme 1:**
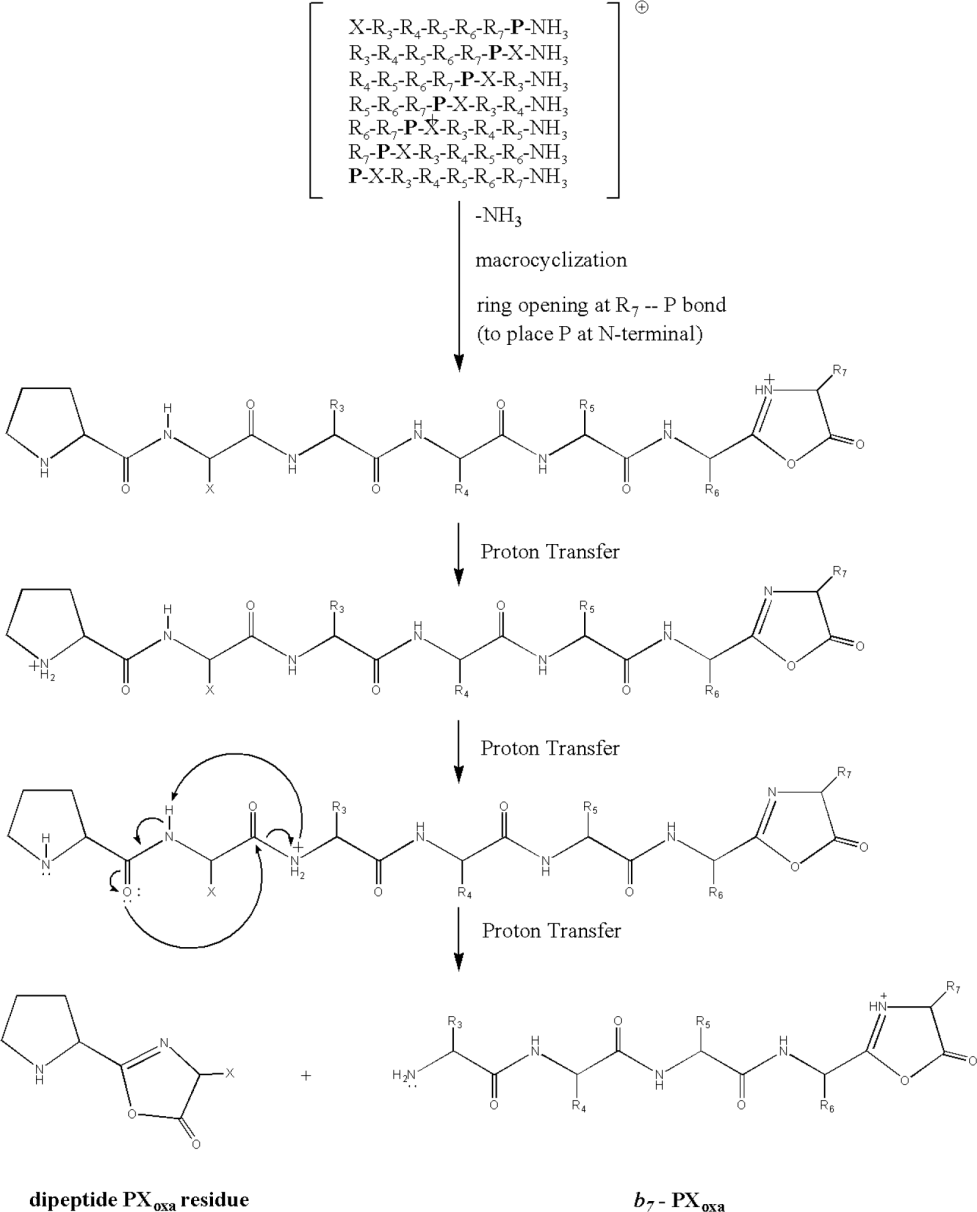
Proposed Reaction Mechanism for Neutral Dipeptide PX Elimination

These results have shown that preferential rearrangement occurs in the peptide sequence during the ring opening of the macrocyclic *b*_7_ structure so that the proline is always located at the N-terminus, no matter its original position in the peptide backbone. The mobile proton is first transferred to the proline pyrrolidine side chain group and then to the second amide bond. This initiates the cleavage of the second amide bond to form a neutral oxazolone dipeptide residue, which explains the PX elimination from the *b*_7_ ion (Scheme 1).

## Conclusions

The current article mainly examines the fragmentation behavior of a series of different model heptapeptides containing a proline residue. The *b*_7_ ions produced from all model isomers of PAAAAAA, PYAGFLV, and AAPXAAA undergo head-to-tail macrocyclization/reopening reactions. Then, a unique fragmentation reaction pathway is observed: neutral proline and its C-terminal residue elimination from the N-terminus of all proline-containing peptides. These abundant dipeptide eliminations from *b*_7_ ions produced from all model heptapeptides suggest that proline-containing *b*_7_ ions tend to place the proline residue in the N-terminal position during the ring opening of the macrocyclic structure which is then followed by elimination of proline plus its adjacent C-terminal residue as neutral *b*_2_ oxazolone (e.g., PX_oxa_). Overall, the obtained results are particularly important, highlighting the unique behavior of proline-containing heptapeptide isomers. This observation can help in the development of new search algorithms to prevent false positives in protein identification.

## Supporting Information Available

Figure S1, CID spectra of *b*_7_ ions from other isomers of the PAAAAAA-NH_2_ series; Figures S2–S6, CID spectra of *b*_7_ ions from PLVYAGF/YAGFPLV, PAGFLVY/YPAGFLV, PGFLVYA/YAPGFLV, PFLVYAG/YAGPFLV, and PYAFLVG/PVLFYAG; Figures S7–S11, comparison of MS^4^ spectra of PX_oxa_ eliminations; Figure S12, CID spectra of *b*_7_ ions from protonated (a) YAGHFLV-NH_2_, (b) YAGKFLV-NH_2_, and (c) YIHPFHL-OH; Figures S13–S18, comparison of CID spectra of *b*_7_ ions from (a) AAPXAAA-NH_2_ and (b) AAXPAAA-NH_2_; Figure S19, CID spectra of *b*_7_ ions from (a) PPAAAAA-NH_2_, (b) AAPPAAA-NH_2_, and (c) AAAAAPP-NH_2_ (PDF)
