# Supplementary material for: Gas Phase Fragmentation Behavior of Proline in Macrocyclic b7 Ions
Source: J Am Soc Mass Spectrom. 2023 Jul 4;34(8):1576–83. doi: 10.1021/jasms.3c00049 (PMC10401712; doi:10.1021/jasms.3c00049)

Supporting Information

Gas Phase Fragmentation Behaviour of Proline in Macrocyclic b_7 Ions

Cagdas Tasoglu[1], Alper Arslanoglu[2]*, Talat Yalcin[1]*

[1] Department of Chemistry, Faculty of Science, Izmir Institute of Technology, Urla-Izmir 35430 Turkey

[2] Department of Molecular Biology and Genetics, Izmir Institute of Technology, Urla-Izmir 35430 Turkey

*Corresponding authors: talatyalcin@iyte.edu.tr and alperarslanoglu@iyte.edu.tr

Contents

Supporting Figures 3

Figure S1. CID spectra of b_7 ion from protonated (a) APAAAAA-NH_2, (b) AAPAAAA-NH_2, (c) AAAAPAA-NH_2, (d) AAAAAPA-NH_2 3

Figure S2. CID spectra of b_7 ions from protonated (a) PLVYAGF-NH_2, (b) YAGFPLV-NH_2 4

Figure S3. CID spectra of b_7 ions from protonated (a) PAGFLVY-NH_2, (b) YPAGFLV-NH_2 5

Figure S4. CID spectra of b_7 ions from protonated (a) PGFLVYA-NH_2, (b) YAPGFLV-NH_2 6

Figure S5. CID spectra of b_7 ions from protonated (a) PFLVYAG-NH_2, (b) YAGPFLV-NH_2 7

Figure S6. CID spectra of b_7 ions from protonated (a) PYAFLVG-NH_2, (b) PVLFYAG-NH_2 8

Figure S7. Comparison of MS^4 spectra of (a) PA_{oxa} elimination from b_7 of protonated PAGFLVY-NH_2 and (b) b_5 of protonated GFLVYA-NH_2 9

Figure S8. Comparison of MS^4 spectra of (a) PG_{oxa} elimination from b_7 of protonated PGFLVYA-NH_2 and (b) b_5 of protonated FLVYAG-NH_2 10

Figure S9. Comparison of MS^4 spectra of (a) PF_{oxa} elimination from b_7 of protonated PFLVYAG-NH_2 and (b) b_5 of protonated LVYAGF-NH_2 11

Figure S10. Comparison of MS^4 spectra of (a) PL_{oxa} elimination from b_7 of protonated PLVYAGF-NH_2 and (b) b_5 of protonated VYAGFL-NH_2 12

Figure S11. Comparison of MS^4 spectra of (a) PV_{oxa} elimination from b_7 of protonated PVYAGFL-NH_2 and (b) b_5 of protonated YAGFLV-NH_2 13

Figure S12. CID spectra of b_7 ions from protonated (a) YAGHFLV-NH_2, (b) YAGKFLV-NH_2, (c) YIHPFHL-OH 14

Figure S13. Comparison of CID spectra of b_7 ions from protonated (a) AAPCAAA-NH_2 and (b) AACPAAA-NH_2 15

Figure S14. Comparison of CID spectra of b_7 ions from protonated (a) AAPDAAA-NH_2 and (b) AADPAAA-NH_2 16

Figure S15. Comparison of CID spectra of b_7 ions from protonated (a) AAPFAAA-NH_2 and (b) AAFPAAA-NH_2 17

Figure S16. Comparison of CID spectra of b_7 ions from protonated (a) AAPGAAA-NH_2 and (b) AAGPAAA-NH_2 18
Figure S17. Comparison of CID spectra of b_7 ions from protonated (a) AAPLAAA-NH_2 and (b) AALPAAA-NH_2 19
Figure S18. Comparison of CID spectra of b_7 ions from protonated (a) AAPVAAA-NH_2 and (b) AAVPAAA-NH_2 20
Figure S19. CID spectra of b_7 ions from protonated (a) PPAAAAA-NH_2, (b) AAPPAAA-NH_2, (c) AAAAAPP-NH_2 21

Supporting Figures

Figure S1. CID spectra of b_7 ion from protonated (a) APAAAAA-NH$_2$, (b) AAPAAAA-NH$_2$, (c) AAAAPAA-NH$_2$, (d) AAAAAPA-NH$_2$

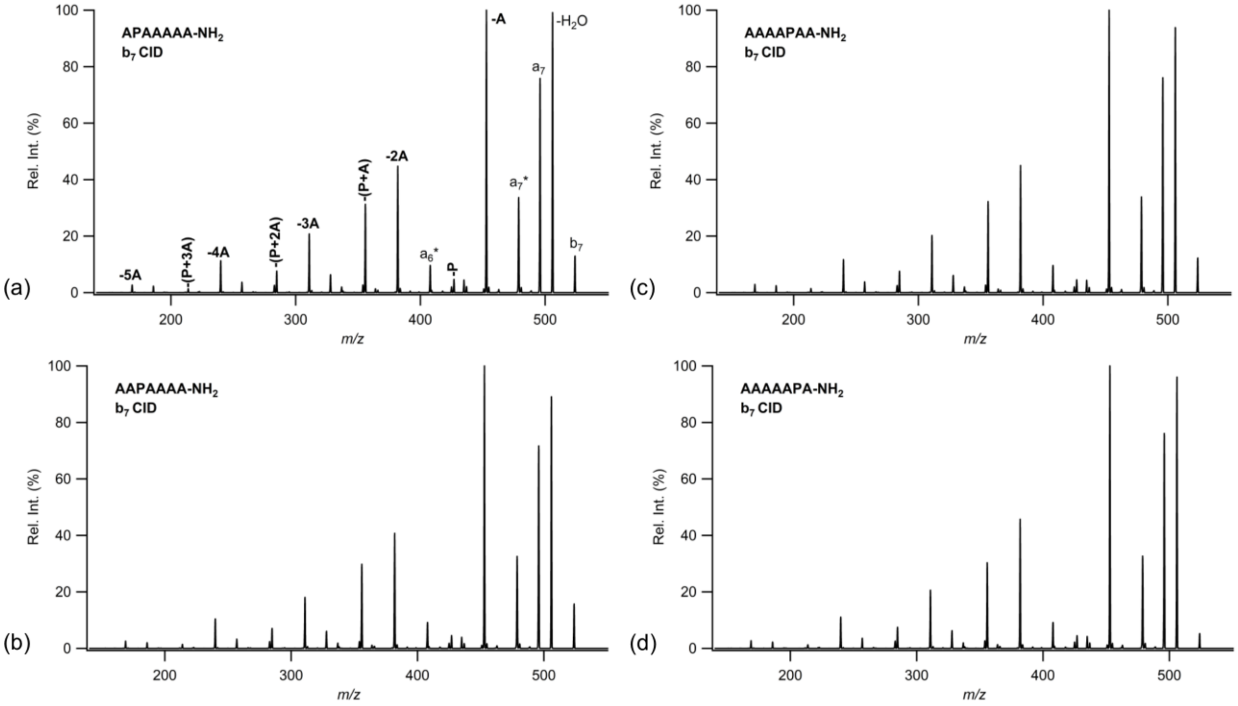

Figure S2. CID spectra of b_7 ions from protonated (a) PLVYAGF-NH_2, (b) YAGFPLV-NH_2

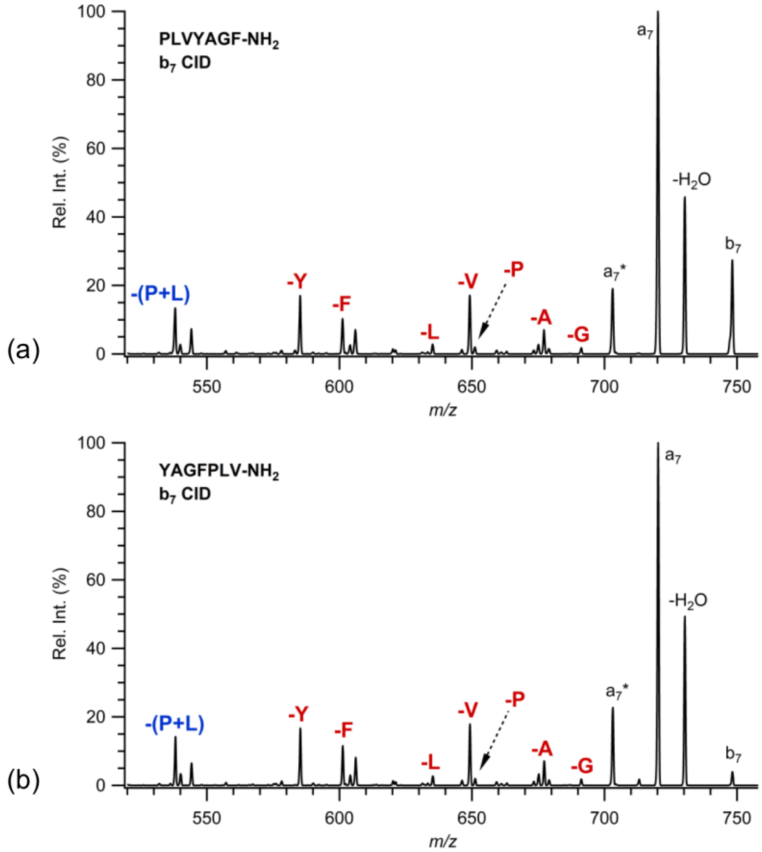

Figure S3. CID spectra of b_7 ions from protonated (a) PAGFLVY-NH_2, (b) YPAGFLV-NH_2

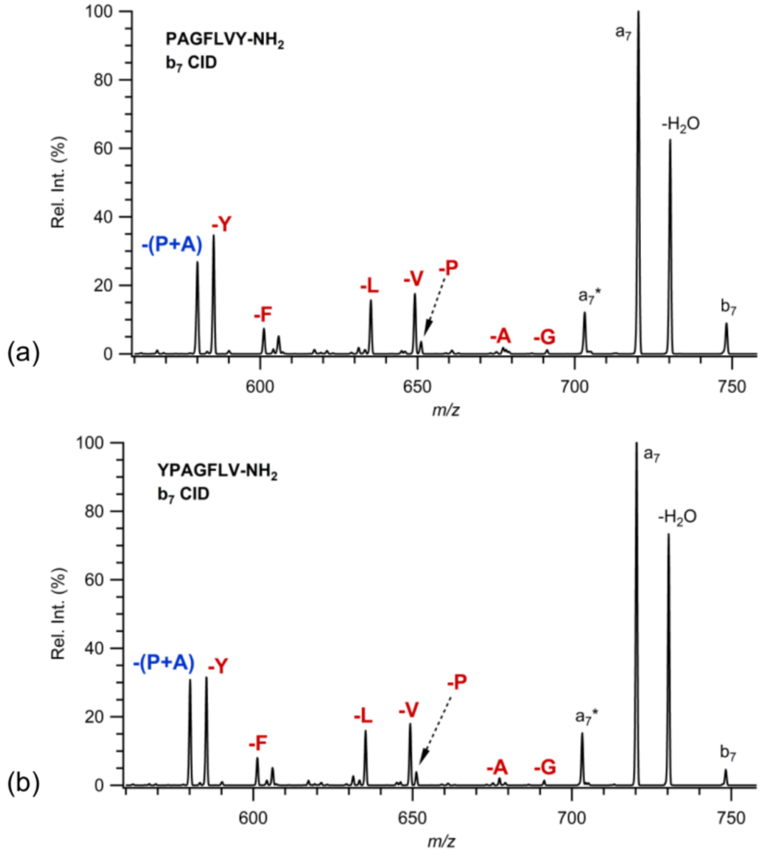

Figure S4. CID spectra of b_7 ions from protonated (a) PGFLVYA-NH_2, (b) YAPGFLV-NH_2

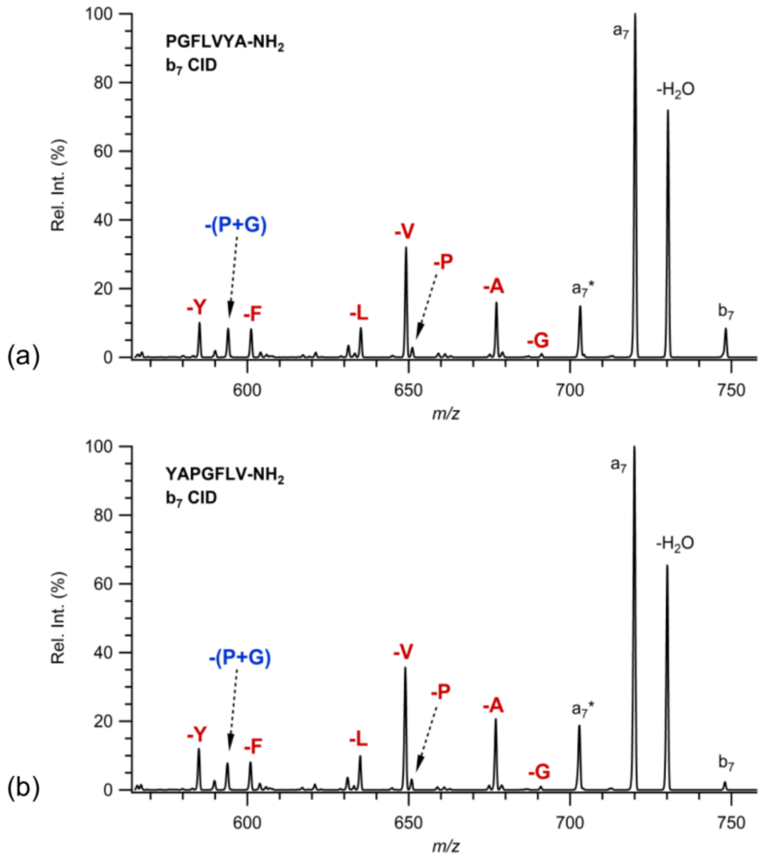

Figure S5. CID spectra of b_7 ions from protonated (a) PFLVYAG-NH_2, (b) YAGPFLV-NH_2

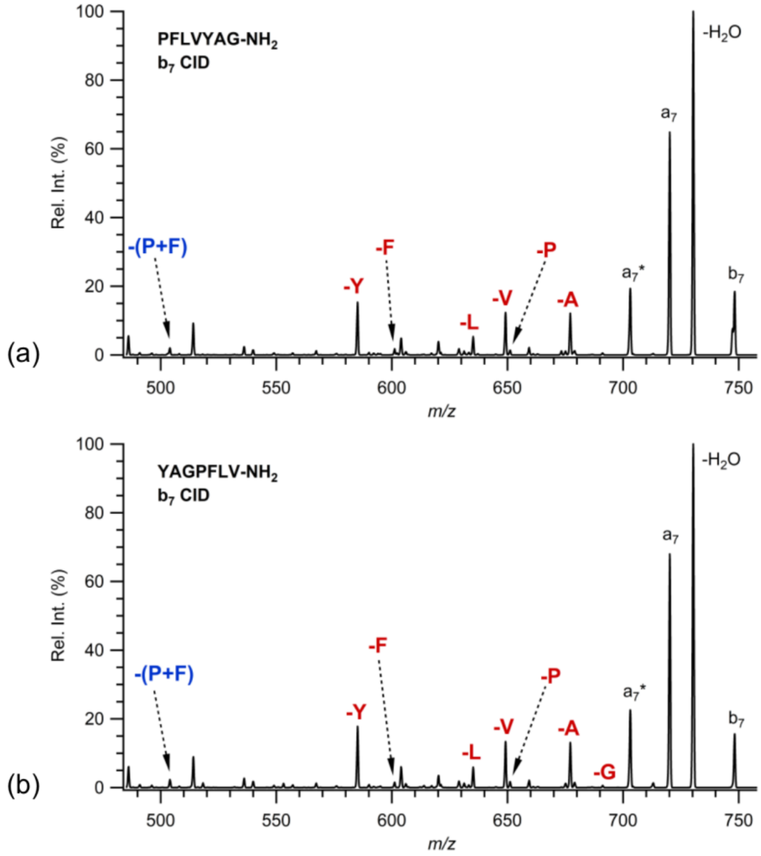

Figure S6. CID spectra of b_7 ions from protonated (a) PYAFLVG-NH_2, (b) PVLFYAG-NH_2

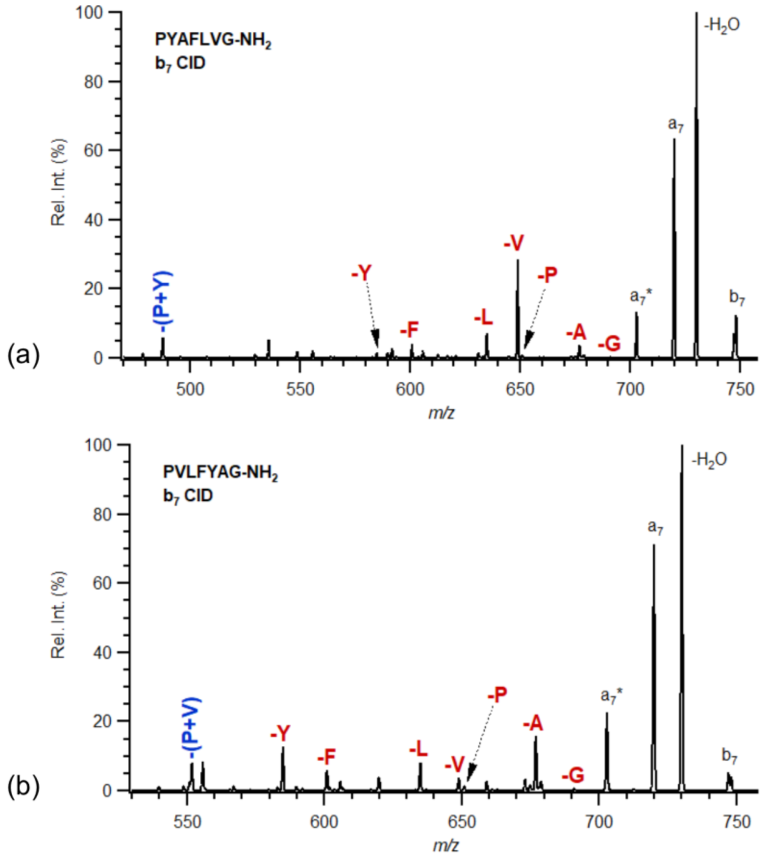

Figure S7. Comparison of MS^4 spectra of (a) PA_{oxa} elimination from b_7 of protonated PAGFLVY-NH_2 and (b) b_5 of protonated GFLVYA-NH_2

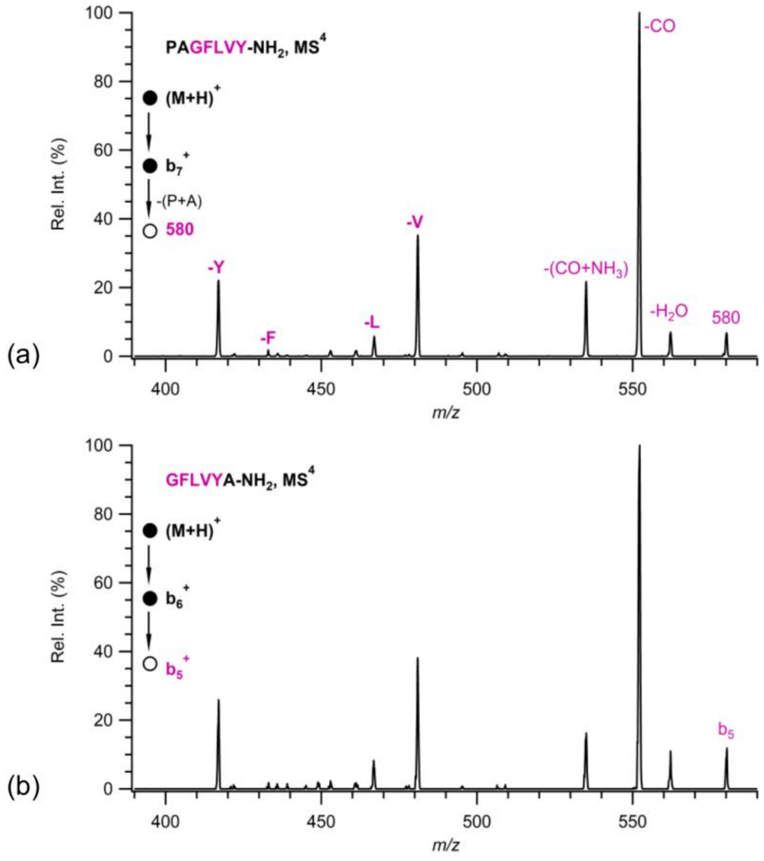

Figure S8. Comparison of MS^4 spectra of (a) PG_{oxa} elimination from b_7 of protonated PGFLVYA-NH_2 and (b) b_5 of protonated FLVYAG-NH_2

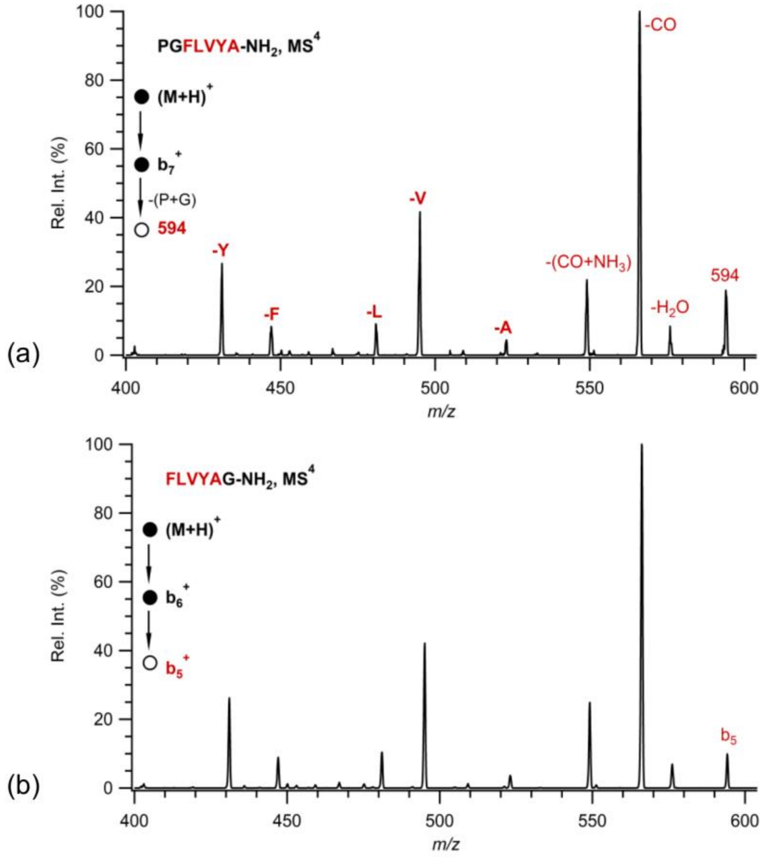

Figure S9. Comparison of MS^4 spectra of (a) PF_{oxa} elimination from b_7 of protonated PFLVYAG-NH_2 and (b) b_5 of protonated LVYAGF-NH_2

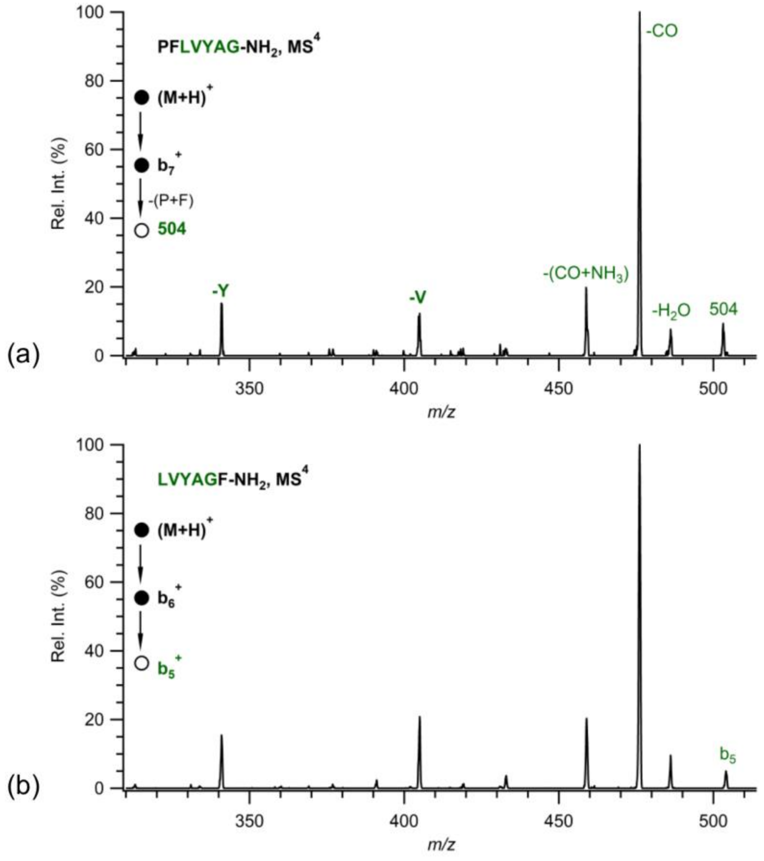

Figure S10. Comparison of MS^4 spectra of (a) PL_{oxa} elimination from b_7 of protonated PLVYAGF-NH_2 and (b) b_5 of protonated VYAGFL-NH_2

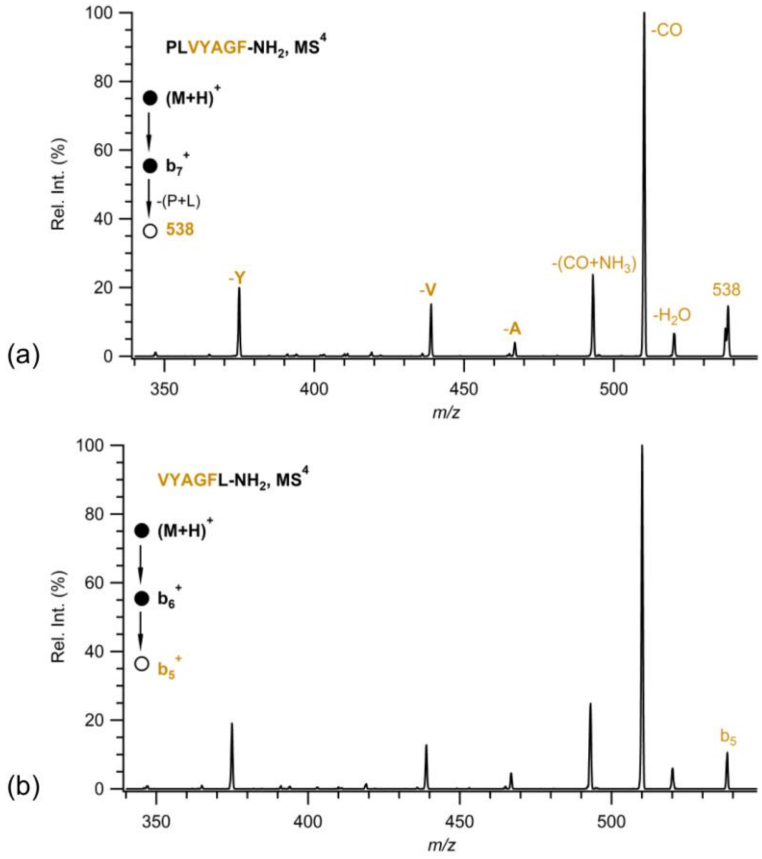

Figure S11. Comparison of MS^4 spectra of (a) PV_{oxa} elimination from b_7 of protonated PVYAGFL-NH_2 and (b) b_5 of protonated YAGFLV-NH_2

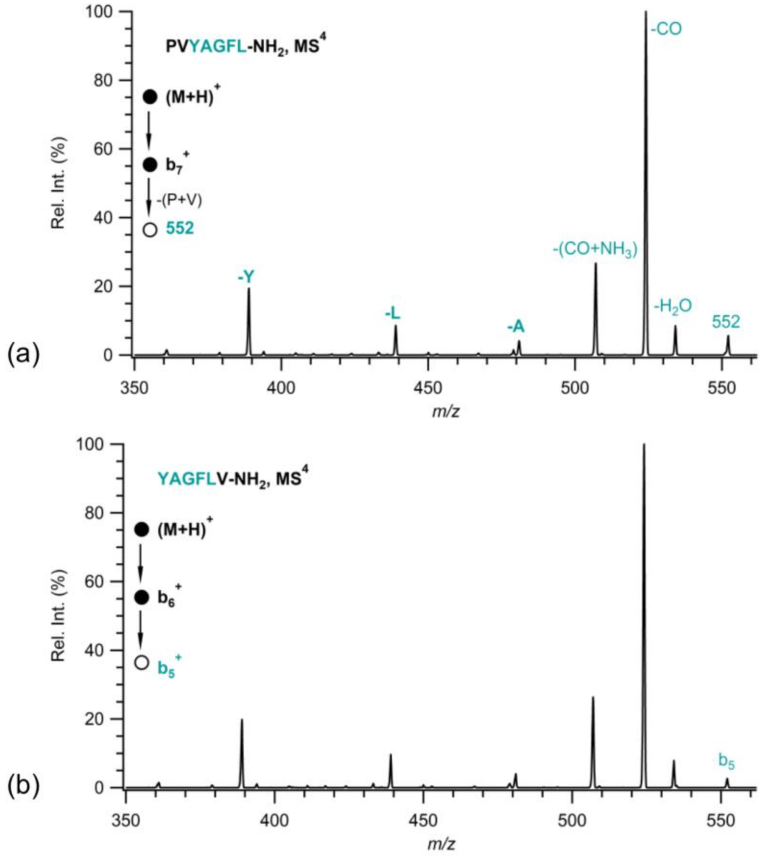

Figure S12. CID spectra of b_7 ions from protonated (a) YAGHFLV-NH_2, (b) YAGKFLV-NH_2, (c) YIHPFHL-OH

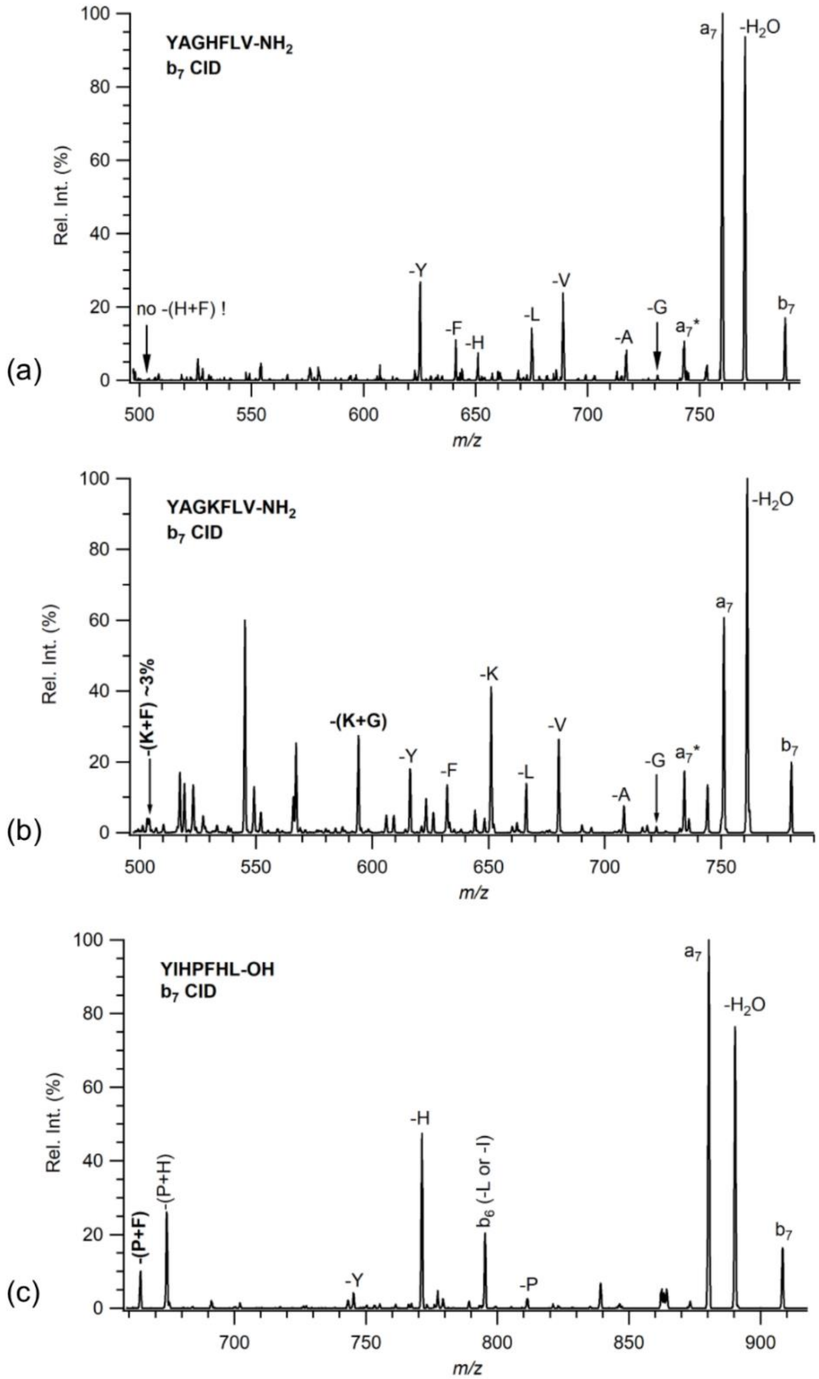

Figure S13. Comparison of CID spectra of b_7 ions from protonated (a) AAPCAAA-NH_2 and (b) AACPAAA-NH_2

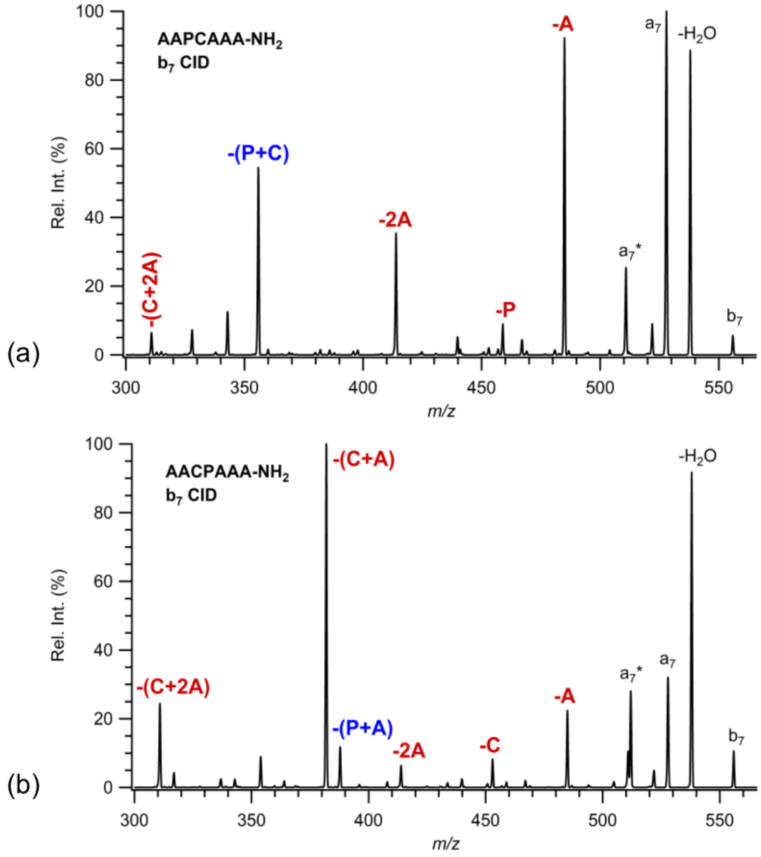

Figure S14. Comparison of CID spectra of b_7 ions from protonated (a) AAPDAAA-NH_2 and (b) AADPAAA-NH_2

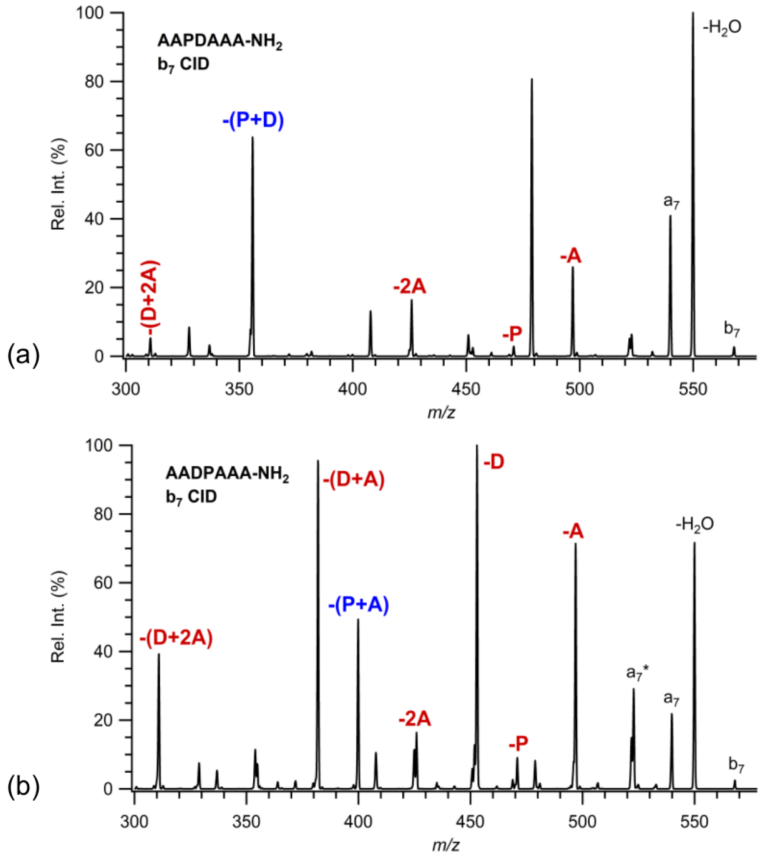

Figure S15. Comparison of CID spectra of b_7 ions from protonated (a) AAPFAAA-NH_2 and (b) AAFPAAA-NH_2

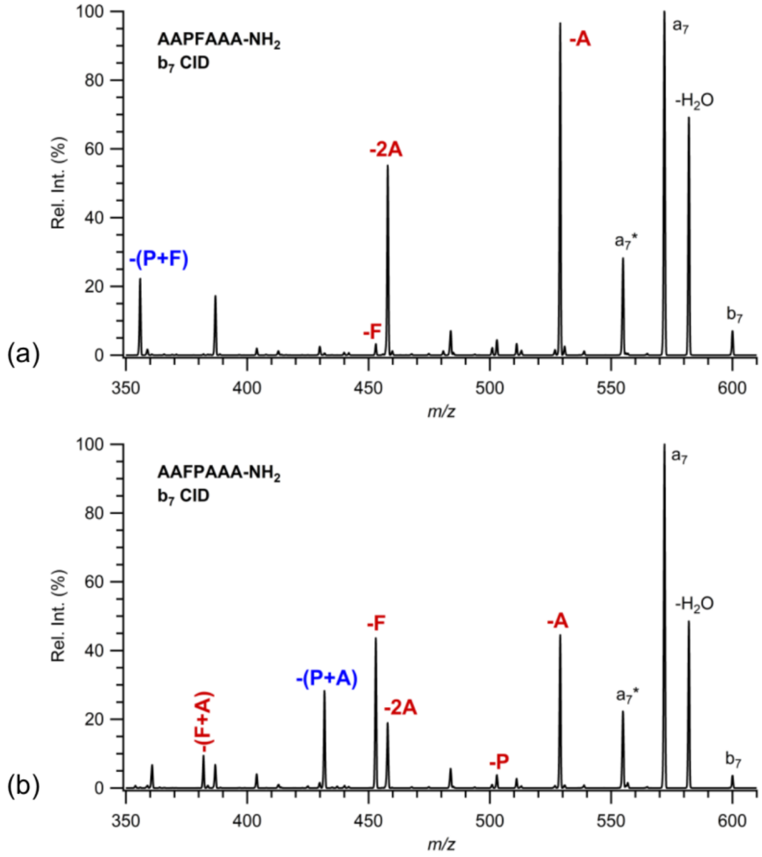

Figure S16. Comparison of CID spectra of b_7 ions from protonated (a) AAPGAAA-NH_2 and (b) AAGPAAA-NH_2

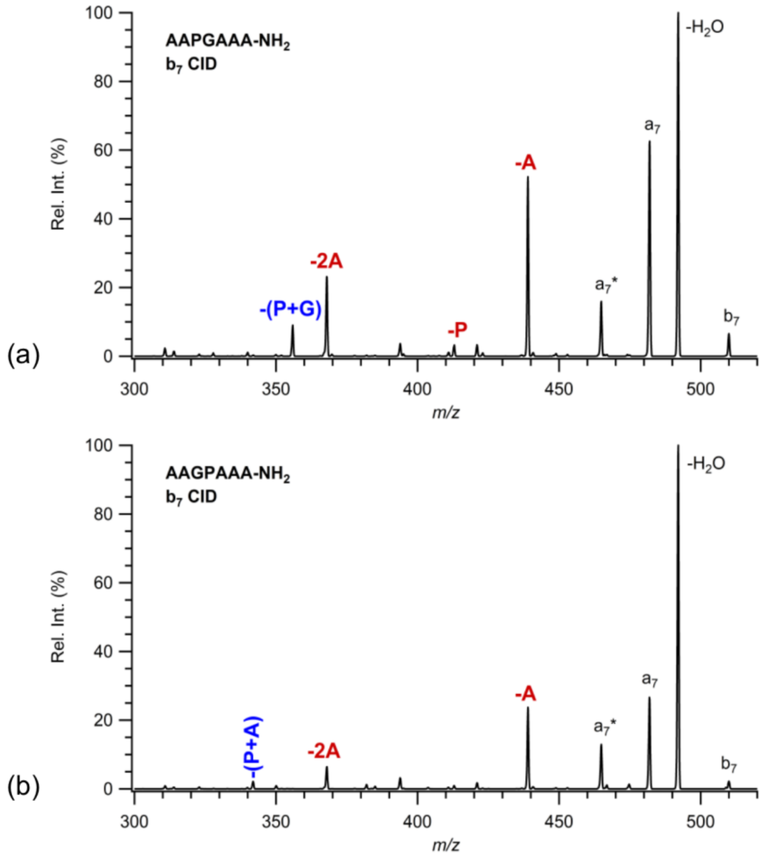

Figure S17. Comparison of CID spectra of b_7 ions from protonated (a) AAPLAAA-NH_2 and (b) AALPAAA-NH_2

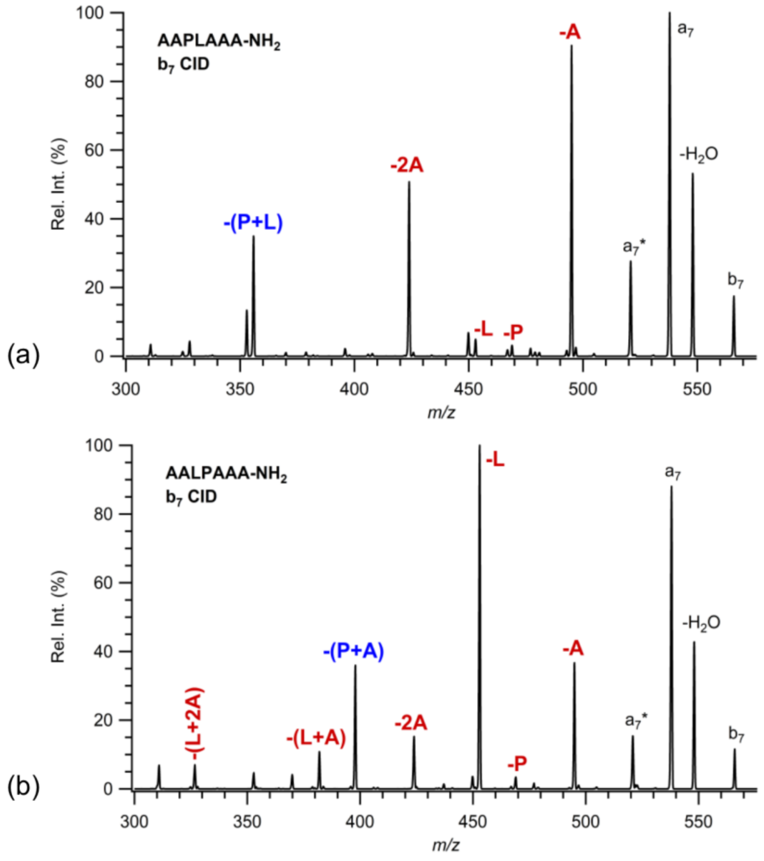

Figure S18. Comparison of CID spectra of b_7 ions from protonated (a) AAPVAAA-NH_2 and (b) AAVPAAA-NH_2

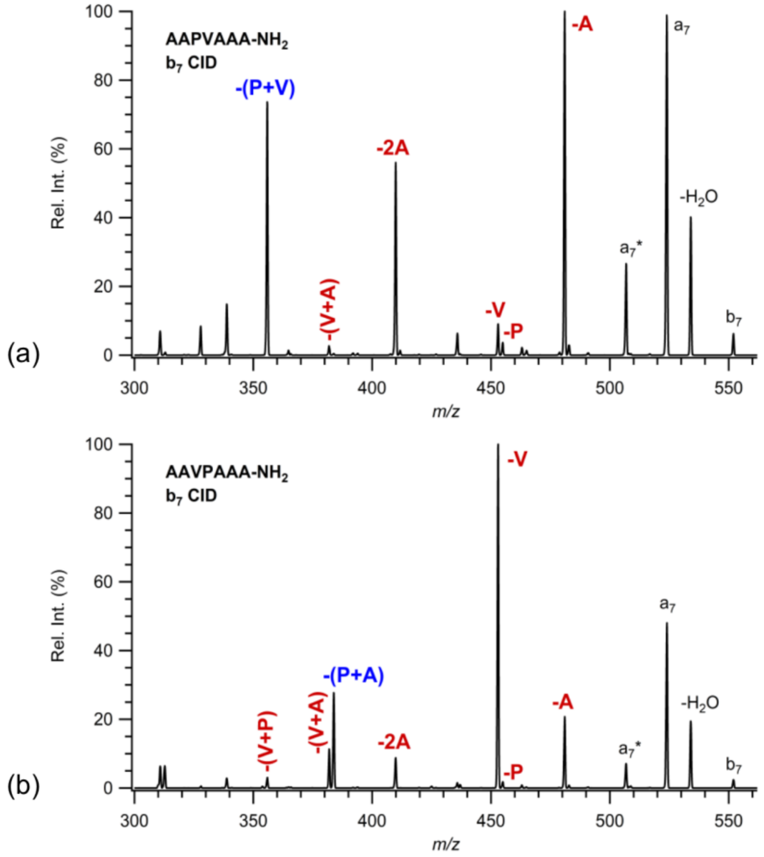

Figure S19. CID spectra of b_7 ions from protonated (a) PPAAAAA-NH_2, (b) AAPPAAA-NH_2, (c) AAAAAPP-NH_2

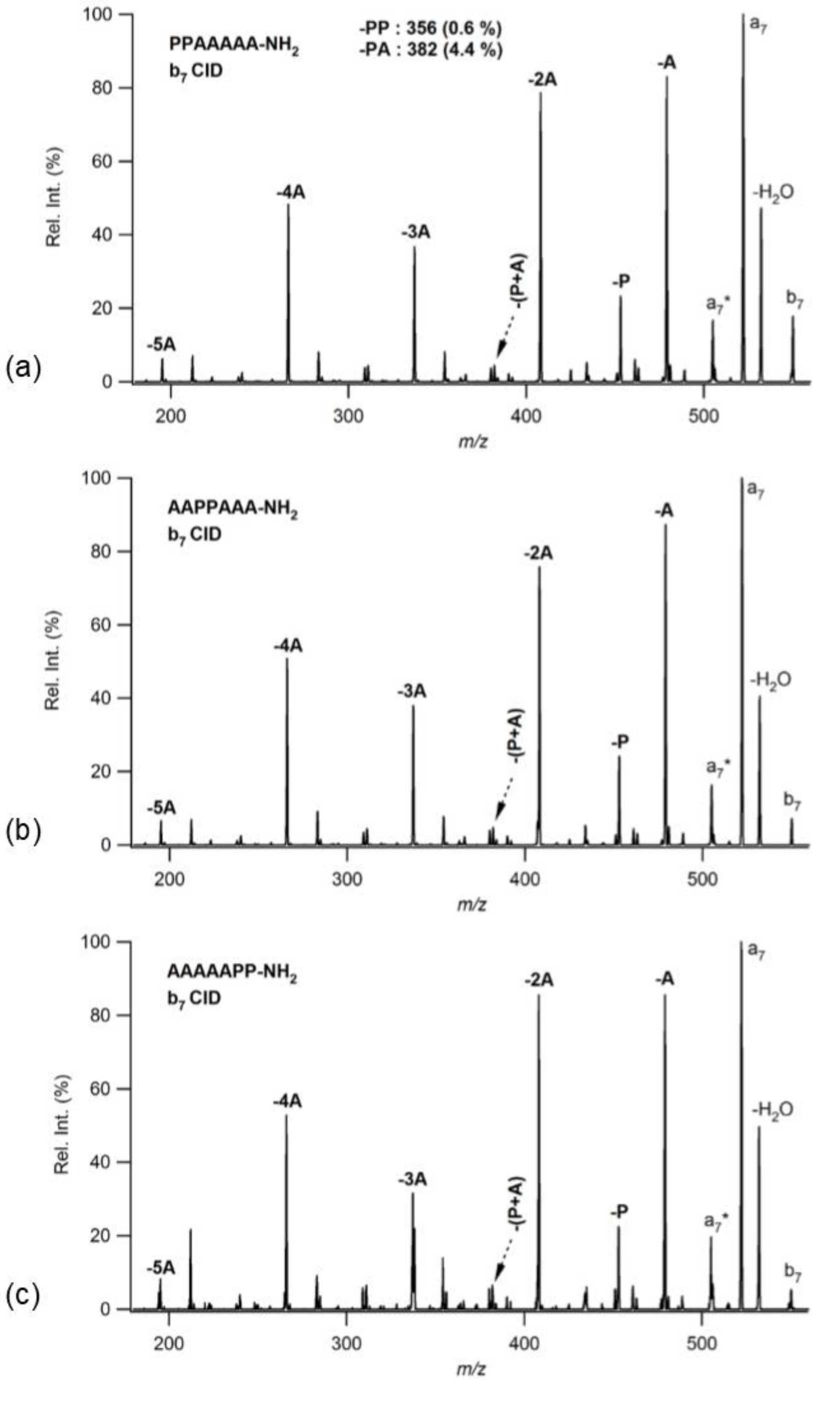

Supplement: Supplementary file 1 — js3c00049_si_001.pdf [file js3c00049_si_001.pdf]
